# Recruitment of Captive‐Reared Florida Grasshopper Sparrows After Translocation: Age of Release Matters

**DOI:** 10.1002/ece3.71662

**Published:** 2025-06-26

**Authors:** Juan C. Oteyza, Karl E. Miller, Andrew Schumann, Sarah Biesemier, Andrea Sylvia

**Affiliations:** ^1^ Florida Fish and Wildlife Conservation Commission Fish and Wildlife Research Institute Gainesville Florida USA; ^2^ White Oak Conservation Foundation Yulee Florida USA; ^3^ Florida Fish and Wildlife Conservation Commission Kenansville Florida USA

**Keywords:** captive breeding, fledgling, hatch‐year, neuroplasticity, recruitment, translocation

## Abstract

Translocation of animals raised in conservation breeding facilities is frequently used as a conservation tool, but few studies have assessed the age of release that maximizes recruitment post‐translocation. For birds, holding captive‐reared juveniles through their first winter is often believed to increase survival by sheltering them from expected high mortality in the wild. However, extended care in captivity requires time and expense and can be associated with the development of tame behaviors; those costs should be weighed against potential benefits. As part of a strategic conservation program for the endangered Florida grasshopper sparrow ( *Ammodramus savannarum floridanus* ), we released 265 sparrows raised under managed care into the Three Lakes Wildlife Management Area population in Florida during 2019–2021. Thirty‐two of 181 (18%) sparrows released as hatch‐years recruited (i.e., were confirmed to be paired and breeding), whereas only 4 of 84 (5%) sparrows released as second‐years recruited. Logistic regression analysis found support for release age class and mass on recruitment probability, but release age class was the only variable included in all the top three models. Based on the most‐parsimonious model, when mass was held at its mean value (16.47 g), the odds of recruitment for hatch‐year birds were 4.65 times (95% CI: 2.04, 11.75) that of second‐year birds. Despite being translocated only a few weeks before the onset of breeding, second‐year birds failed to recruit into the breeding population at rates comparable to individuals translocated 6–8 months earlier as nutritionally independent fledglings. It is unclear whether low recruitment for older sparrows was the result of higher mortality or dispersal. We discuss potential reasons why younger Florida grasshopper sparrows may be developmentally better suited to adapt to novel environments and recommend more research on the role of neuroplasticity during early learning periods and its influence on translocation outcomes.

## Graphical abstract

We found that a non‐migratory passerine raised in a conservation breeding facility demonstrated higher recruitment when translocated soon after fledging than when translocated as second‐year birds after spending their first winter in captivity. Despite being translocated only a few weeks before the onset of breeding, second‐year birds failed to settle and recruit into the breeding population at rates comparable to individuals translocated 6–8 months earlier as juveniles. We discuss potential reasons why younger Florida grasshopper sparrows appeared to be developmentally better suited to adapt to novel environments and recommend more research on the role of neuroplasticity during early learning periods and its influence on translocation outcomes.

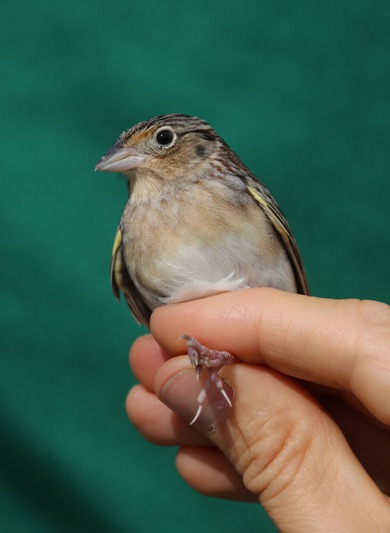

## 1 Introduction

Conservation translocation, the human‐assisted movement of living organisms from one location to another to conserve species and restore ecosystems (IUCN/SSC 2013), is frequently used as a conservation tool but often fails to produce desired outcomes (Fischer and Lindenmayer 2000; Bubac et al. 2019; Berger‐Tal et al. 2020). Organisms for translocation can be sourced from either a wild population or a conservation breeding facility (IUCN/SSC 2013, 2023). Only about 54% of published translocation studies report that translocated organisms were established at release locations, while 21% failed and 25% had unknown outcomes (Bubac et al. 2019). The success of translocation programs can be affected by many factors, including age, sex, number of individuals released, the timing and number of release events, and habitat conditions at the release site, all of which can influence survival, productivity, and dispersal (Fischer and Lindenmayer 2000; Letty et al. 2007; Bennett et al. 2012; Jones and Merton 2012; Parker et al. 2012; Carrlson et al. 2014; Batson et al. 2015; Kemp et al. 2015; Destro et al. 2018). For birds, the age of animals that are translocated may be important in maximizing the likelihood of establishment and population growth (Sarrazin and Legendre 2000; Buner and Schaub 2008; Masuda and Jamieson 2012; VanderWerf et al. 2014). However, potential impacts associated with the age of release are rarely addressed in the literature. Many translocation projects lack comparative data to assess the relative success of different age classes in translocations, limiting our ability to draw unifying conclusions and recommendations for practitioners (Miller et al. 2024).

Translocation projects often source individuals for release from a conservation breeding program. A common goal of many of these programs is to provide a safe environment for individuals during a relatively vulnerable stage of their life when they would face high mortality rates in the wild, effectively providing a head start (Cristinacce et al. 2008; Cunninghame et al. 2015). At the same time, prolonged periods under managed care may have detrimental impacts on an individual's ability to adapt successfully to conditions in the wild after release (Hardouin et al. 2014; Berger‐Tal et al. 2020; Tripovich et al. 2021; Wu et al. 2024). Therefore, the success of a conservation breeding and release program requires determining the age of release that optimizes the trade‐off between increasing survival and maintaining wild behaviors.

For passerines, the decision of when to release birds bred under managed care can be influenced by two periods of high mortality in the wild: during the post‐fledging period when birds are still dependent on their parents (Streby and Andersen 2011; Cox et al. 2014) and during their first winter, when unfavorable weather conditions or food shortages may occur (Newton 1998). Consequently, release into the wild may be timed to occur after these stressors, either as soon as birds become nutritionally independent from their parents, or at the end of their first winter, just prior to the breeding season (Batson et al. 2015). However, few studies have assessed the relative impacts of these two approaches on subsequent recruitment, and typically without conclusive results (Hameau and Millon 2019; Tripovich et al. 2021; Miller et al. 2024).

As part of a strategic conservation program for the Florida grasshopper sparrow ( *Ammodramus savannarum floridanus* ), we conducted releases of two age classes within an experimental and adaptive framework (Kemp et al. 2015). The Florida grasshopper sparrow is a non‐migratory subspecies endemic to dry‐prairie habitat in central Florida. It is federally listed as endangered under the U.S. Endangered Species Act and is critically imperiled (Perkins et al. 2008; Tucker Jr et al. 2010; Delany et al. 2014; Hewett Ragheb, Miller, and Kiltie 2019). In 2018, the year before releases began, only ~25 breeding pairs existed in the wild across all known populations on public and private lands (Florida Grasshopper Sparrow Working Group, unpublished data). Our objectives were (1) to release birds as independent juveniles (i.e., hatch‐years) during the summer and as young adults (i.e., second‐years) after their first winter, (2) to closely monitor the population at the release site during subsequent breeding seasons to determine recruitment rates (the number of released birds that became breeders), and (3) to identify the effects of release age, sex, and body condition on recruitment.

## 2 Methods

### 2.1 Study Design and Cohorts

**FIGURE 1:**
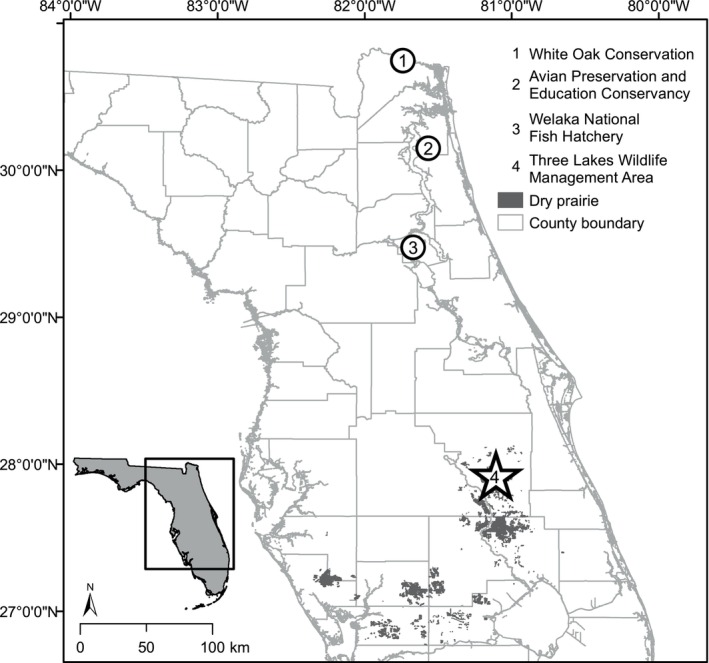
Location of primary conservation breeding facility (White Oak Conservation), additional breeding and holding facilities (Avian Preservation and Education Conservancy, Welaka National Fish Hatchery), and recipient population (Three Lakes Wildlife Management Area) for the Florida grasshopper sparrow release experiment, 2019–2022, Florida, USA.

We released all sparrows at Three Lakes Wildlife Management Area (hereafter, Three Lakes), which is located near the town of Kenansville (27.8768° N, 80.9888° W) in Osceola County, Florida, USA (Figure 1). Native dry prairie (Abrahamson and Hartnett 1990) at Three Lakes is interspersed with patches of wet prairie and depression ponds and maintained in a treeless condition by prescribed fire (Hewett Ragheb, Miller, and Kiltie 2019) and occasional mechanical disturbance. Vegetation is predominantly wiregrass (*Aristida* spp.), bluestem (*Andropogon* spp.), stunted (< 0.6 m tall) saw palmetto ( *Serenoa repens* ), and dwarf live oak ( *Quercus minima* ; Florida Natural Areas Inventory 2009; Larned et al. 2020). Florida grasshopper sparrows were first described based on a specimen collected in 1901 by Mearns (1902) on or near present‐day Three Lakes (Pranty and Tucker Jr 2006). The study area has been continuously occupied by Florida grasshopper sparrows since at least the early 1980s (USFWS 1986), monitored since 1991 (Tucker Jr et al. 2010; Delany et al. 2013, 2014; Hewett Ragheb, Miller, and Kiltie 2019; Hewett Ragheb, Miller, and Leone 2019), and currently supports the largest remaining known population (Florida Grasshopper Sparrow Working Group, unpublished data).

**FIGURE 2:**
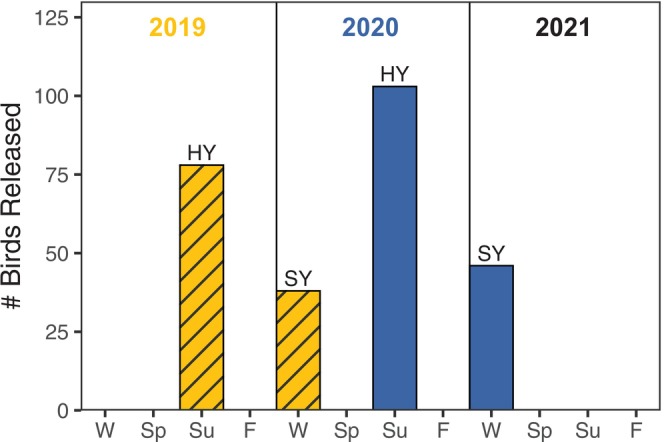
Schematic of temporal study design for translocation of captive‐reared Florida grasshopper sparrows to Three Lakes Wildlife Management Area, Florida, USA at different times throughout the annual cycle (W = winter [mid‐December − mid‐March], Sp = spring [mid‐March – mid‐June], Su = summer [mid‐June – mid‐September], F = fall [mid‐September – mid‐December]). Sparrows from the 2019 cohort (yellow hatched) and 2020 cohort (blue) were released at two different ages (hatch‐year [HY] and second‐year [SY]) and recruitment for all birds was measured in the first spring after their release. Note that some “summer” releases occurred as early as late May.

The release study included two cohorts (2019, 2020) of Florida grasshopper sparrows reared under managed care. Each cohort was split into two different groups: one group was released as hatch‐year juveniles during the summer (late May through early September) after reaching nutritional independence, and one group was released near the end of their first winter (early February through mid‐March) as second‐year birds (Figure 2). Mean age of release for hatch‐year sparrows was 50.0 (±7.6 SD) days post hatching (range: 41–77 days). Mean age of release for second‐year sparrows was 225.1 (±35.6 SD) days post‐hatching (range: 168–305 days). Individual group assignment (into hatch‐year vs. second‐year release) was based on a combination of factors, including hatch date and plumage condition. Birds that hatched earlier in the season were more likely to be released during the summer as hatch‐years. Birds that had plumage irregularities were sometimes held overwinter to go through a molt cycle and then released as second‐years.

### 2.2 Conservation Breeding

Florida grasshopper sparrows were collected by the U.S. Fish and Wildlife Service for breeding starting in 2015 and sourced from three wild populations. Florida grasshopper sparrows were primarily raised in conservation breeding facilities at White Oak Conservation (hereafter White Oak) in Yulee, FL, USA (Figure 1). After successful conservation breeding protocols were developed at White Oak during 2016–2019, additional sparrows were raised nearby at the Avian Preservation and Education Conservancy in Jacksonville, FL, using the same protocols. Ninety‐five percent (248 of 265) of the sparrows released in our experiment were hatched and raised at White Oak. Some sparrows were staged temporarily at Welaka National Fish Hatchery, a second facility located in Welaka, FL, prior to their release at Three Lakes to ease capacity constraints at White Oak.

At the conservation breeding facilities, birds were kept in aviaries and paired up before the breeding season. Pairing decisions were made based on social compatibility and pedigree data to avoid inbreeding and maximize genetic diversity (A. Schumann, unpublished data). All efforts were made to mimic conditions in the wild inside the sparrow enclosures (e.g., providing preferred nesting substrate and ground cover) to facilitate the expression of natural behaviors observed in the wild (Greggor et al. 2018). Adults built nests, laid and incubated eggs, and fed young until fledging without human assistance. Fledglings remained with their parents until ~23 days after hatching, when they become independent and can feed on their own (Vickery 1996; Small et al. 2015; Hewett Ragheb, Miller, and Leone 2019). Independent fledglings were then separated and placed in a new aviary with their siblings and other independent sibling groups, a social structure that mimicked behaviors we observed in the wild. Juveniles were handled on no more than two or three occasions to minimize stress (Parker et al. 2012), including the day they were transferred to the release site. Handling prior to their release was necessary to perform an initial health check, to administer immunizations (West Nile Innovator for prevention of West Nile Virus, Eastern and Western Equine Encephalitis Virus; Zoetis, Parsippany‐Troy Hills, NJ, USA), and to collect a buccal swab for sexing. On the day of transport for release, birds were captured to collect morphometric data (including mass and fat score Wenker et al. 2022), and to apply a numbered USGS aluminum leg band and a unique combination of color plastic leg bands for identification in the wild.

### 2.3 Transport and Release

We placed sparrows in wooden crates (~28 cm × 13 cm × 18 cm) for transport to the release site, with one bird per compartment. Each compartment was large enough to hold a bird comfortably and allow it to move, but small enough to reduce the risk that a bird would try to fly and potentially injure itself during transport. We lined transport crates with wiregrass (*Aristida* sp.) and included mealworm beetle larvae ( *Tenebrio molitor* ). Typically, 6–12 sparrows were transported to the Three Lakes study area during each release event. Once at the release site, we lifted the sliding door of the transport crate, which allowed the birds to exit on their own into a field aviary in dry‐prairie habitat.

We acclimated sparrows from the 2019 cohort in a stationary aviary (6 m × 18.3 m × 2.4 m) and sparrows from the 2020 cohort in either the stationary aviary or a mobile aviary (2 m × 4.5 m × 2 m) built on a dual‐axle utility trailer. Ninety‐five percent (252 of 265) of the sparrows released in our experiment were acclimated using the stationary field aviary. Time in the aviaries allowed birds to consume food and water (i.e., seeds, insects, and insect larvae provided at feeding stations) and recover from transport. Motion‐sensor activated cameras (www.moultrie.com; Birmingham, AL, USA) were set at the feeding stations and the footage checked daily to confirm that birds were behaving normally and eating. After 1–3 nights of acclimation in the aviary, we removed the side panels of the enclosures in the morning and allowed the birds to exit. If any birds remained inside the enclosure after a 30–60‐min period, we walked inside the aviary to gently flush them out.

### 2.4 Breeding Season Monitoring

For this experiment, our measure of translocation success was the successful recruitment of sparrows into the breeding population. Florida grasshopper sparrows are cryptic birds that are very difficult to detect outside of the breeding season, even with invasive methods like sweep‐nest sampling (Korosy et al. 2013). Therefore, we conducted no monitoring during fall and winter to minimize disturbance. We determined a released sparrow had survived and recruited into the breeding population when it was resighted on a breeding territory and confirmed to be paired and nesting during April–August in conjunction with an ongoing demographic study at Three Lakes (Hewett Ragheb, Miller and Kiltie 2019; Hewett Ragheb, Miller, and Leone 2019). Evidence of a nesting attempt was an important criterion for females, who are sometimes detected and identified only after nests are located (Hewett Ragheb and Miller 2016).

We followed long‐established protocols to detect Florida grasshopper sparrows, determine their breeding status, and find and monitor their nests during April–August 2019–2022 on a ~ 3000 ha patch of dry‐prairie habitat at Three Lakes (Hewett Ragheb and Miller 2016; Hewett Ragheb, Miller and Kiltie 2019; Hewett Ragheb, Miller, and Leone 2019). Adult male Florida grasshopper sparrows are highly philopatric across years (Dean et al. 1998) and defend small territories (average 1.8 ha; Delany et al. 1995) throughout the breeding season (March–August). We performed systematic point‐count surveys on established grids (Delany et al. 2013) at the release site throughout the breeding season. Once birds were detected, we performed behavioral observations to determine if birds were paired or if males were simply holding a territory. Florida grasshopper sparrow males change their song when paired with a female (Vickery 1996; Lohr et al. 2013). If paired, we searched for their nest. Florida grasshopper sparrows build concealed, domed nests on the ground (Delany and Linda 1998; Larned et al. 2021). Once a nest was found, we placed predator deflection fences around the nest to reduce mammalian predation and installed a nest camera to help determine the fate of the nest (Hewett Ragheb, Miller, and Leone 2019). Pairs can renest throughout the breeding season (Hewett Ragheb, Miller, and Kiltie 2019), with each nesting cycle 19–22 days from the start of incubation to fledging (Vickery 1996). Age at first breeding is typically second‐year (i.e., the first spring after hatching; Vickery 1996). We considered a captive‐reared sparrow to have successfully recruited if it was confirmed to be paired and breeding (i.e., nesting). In our color‐banded sparrow population, breeding territories rarely go undetected, and few nests fledge without our knowledge (Hewett Ragheb and Miller 2016; Hewett Ragheb, Miller, and Leone 2019).

### 2.5 Analysis

We fit logistic regression models in program R (version 4.3.0; R Core Team 2023) to evaluate the influence of factors affecting the recruitment probability of released sparrows. Nearly all individuals that were recruited did so in the first breeding season following their release (Table 1). For consistency in analyses, we used recruitment data from the first breeding season post‐release to compare results between cohorts and age classes. Models included the independent variables, release age class (hatch‐year or second‐year), cohort (2019 or 2020), sex, mass, acclimation period length, and fat scores. We fit 16 models describing additive combinations of independent variables. We did not include interactive models as they were poorly estimated in preliminary steps, likely due to the low number of detections in some categories. We used model selection based on Akaike's Information Criterion for small sample sizes (AIC_c_; Akaike 1973; Hurvich and Tsai 1989) where models with lower AIC_c_ values and higher Akaike weights are considered the most parsimonious. The best‐approximating set of models were considered those with summed Akaike weights totaling at least 0.95 (Burnham and Anderson 2002). Model fit was confirmed through residual diagnostics on scaled residuals using a simulation approach with the DHARMa package (Hartig 2022). Predictive performance of the top supported models was evaluated by assessing the in‐sample bootstrapped area under the receiver operator curve (AUROC) using the ROCR package (Sing et al. 2005). The AUROC assesses the model's ability to correctly classify the dependent variable, where a value of 0.50 indicates discrimination based on random chance and a value of 1.0 represents perfect discrimination. We considered variables whose 95% bootstrapped confidence intervals did not overlap zero to significantly describe variation in recruitment.

**TABLE 1:** Cohorts of captive‐reared Florida grasshopper sparrows ( *Ammodramus savannarum floridanus* ) released at Three Lakes Wildlife Management Area, FL, USA during 2019–2022, with the number (and percentages) that recruited from each age class for release (HY = hatch‐year, SY = second‐year).

Cohort	Release age class	*N*	Recruitment	
			1st year post‐release	1st and 2nd year post‐release
2019	HY	78	16 (21%)	19 (24%)
	SY	38	0 (0%)	1 (3%)
2020	HY	103	16 (16%)	16 (16%)
	SY	46	4 (9%)	4 (9%)
Cohorts combined	HY	181	32 (18%)	35 (19%)
	SY	84	4 (5%)	5 (6%)

*Note:* Results are presented for the first year post‐release and the first two years post‐release, but only results from the first year post‐release were included in analyses.

During the first year of our study, we tagged a subset of released birds (*n* = 17 hatch‐years, 11 s‐years) with VHF radio transmitters (Lotek, Ontario, Canada) for a companion study. Most tagged sparrows were tracked for less than a week before the tags stopped transmitting or the birds disappeared (J. Oteyza and K. Miller, unpublished data). As a precaution, we used logistic regression to fit an additive model of the presence of transmitter and release age class and confirmed model fit through residual diagnostics (Hartig 2022). The presence of a transmitter did not influence recruitment rates (*β*_tag_ = −0.10 [95% CI: −1.14, 0.80]) of grasshopper sparrows, so we did not include it as a variable in subsequent analyses.

## 3 Results

We translocated a total of 265 Florida grasshopper sparrows raised under managed care into the Three Lakes population during 2019–2021. All birds were safely translocated to the release site and released without incident. The 2019 cohort included 78 hatch‐year (i.e., juvenile) releases and 38 second‐year (i.e., young adult) releases, and the 2020 cohort included 103 hatch‐year releases and 46 second‐year releases (Figure 2, Table 1). Thirty‐six of the 265 Florida grasshopper sparrows (13.6%) were confirmed breeders (recruited) at Three Lakes during the first breeding season after their release (Table 1). A total of 32 hatch‐years (17 males, 15 females) recruited, whereas only 4 second‐years (three males, one female) recruited.

Logistic regression analysis confirmed that recruitment differed between release age classes. Model results indicated support for release age class, mass, and sex on the recruitment probability of grasshopper sparrows, but release age class was the only variable included in all of the top three models (Table 2). The top supported model included additive effects of release age class, mass, and sex and was 2.2 times more plausible than the second supported model, which included the additive effects of release age and mass (Table 2). However, the effect of sex was imprecise in the top supported model, with confidence intervals that overlapped zero (Table 3), indicating that it was an uninformative parameter (Arnold 2010). Therefore, we selected the second‐most supported model with additive effects of release age class and mass as our best model and the more parsimonious model.

**TABLE 2:** Logistic regression model set results used to determine the best‐approximating models describing the probability of recruitment of captive‐reared Florida grasshopper sparrows released at Three Lakes Wildlife Management Area, FL, USA during 2019–2021.

Model	*K*	AIC_c_	ΔAIC_c_	W_i_
Age class at release + mass + sex	4	258.97	0.00	0.44
Age class at release + mass	3	260.54	1.57	0.20
Age class at release + sex	3	261.16	2.19	0.15
Age class at release + sex + fat reserves	4	261.80	2.83	0.11
Age class at release + mass + fat reserves	4	262.36	3.39	0.08
Age class at release	2	266.45	7.48	0.01
Age class at release + fat reserves	3	267.55	8.58	0.01
Age class at release + annual cohort	3	267.57	8.60	0.01
Age class at release + acclimation period	3	268.04	9.07	0.01
Sex	2	268.77	9.80	0.00
Mass + Sex	3	270.59	11.62	0.00
Intercept	1	272.68	13.71	0.00
Mass	2	272.99	14.02	0.00
Acclimation period	2	273.62	14.65	0.00
Annual cohort	2	273.92	14.95	0.00
Fat reserves	2	274.12	15.15	0.00

*Note:* Parameters in the table include ∆AIC = relative difference from the best model, AIC = Akaike's Information Criterion, *K* = number of parameters and Wi = Akaike weight.

**TABLE 3:** Parameter estimates, standard errors (SE), 95% confidence intervals (95% CI), odds ratios (OR), and area under the receiver operator curve (AUROC) for the top supported models (as determined by AIC_c_ scores; see Table 2) describing the probability of recruitment of captive‐reared Florida grasshopper sparrows released at Three Lakes Wildlife Management Area, FL, USA during 2019–2021.

Parameter	AUROC score	Estimate	SE	95% CI	OR
*Best‐approximating model*	0.69				
Intercept		−8.37	2.81	−13.99, −2.93	
Age class at release		1.52	0.54	0.68, 2.45	4.55
Mass		0.33	0.16	0.02, 0.65	1.40
Sex		0.65	0.35	−0.02, 1.35	1.92
*Second‐best approximating model*	0.67				
Intercept		−9.53	2.71	−14.99, −4.31	
Age class at release		1.54	0.44	0.71, 2.47	4.65
Mass		0.43	0.15	0.13, 0.73	1.53

*Note:* Age class at release coded as 1 = hatch year, 0 = second year; sex coded as 1 = male, 0 = female. AUROC describes model performance and prediction accuracy, where a value of 1.0 is perfect. Sex was an uninformative parameter in the best‐approximating model; therefore, our conclusions are based on the second‐best approximating model, which is also the most‐parsimonious model.

**FIGURE 3:**
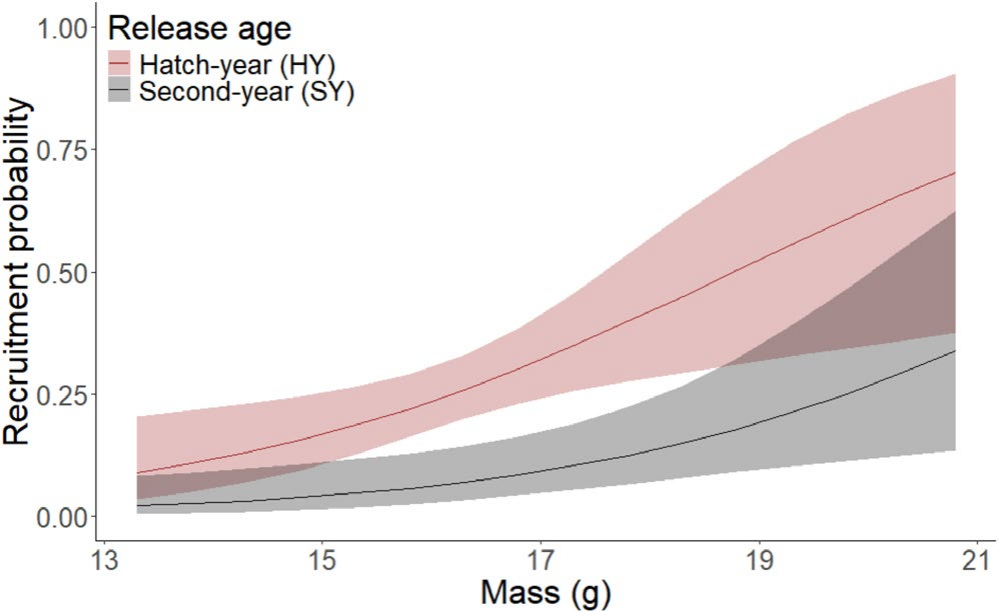
Recruitment probability with increasing mass for Florida grasshopper sparrows released at Three Lakes Wildlife Management Area, Florida, USA as either hatch‐year (HY: Red) or second‐year (SY: Gray). Predictions derived from the most‐parsimonious model (see Table 3). Lines represent the mean predictions, and shaded areas represent the 95% confidence intervals around predictions.

Based on the best model, the odds of recruitment for hatch‐year sparrows when mass was held at its mean value (16.47 g) were 4.65 times (95% CI: 2.04, 11.75) that of sparrows released as second years (Table 3). Recruitment also was associated with mass, where for every 1 g increase in mass, recruitment was 1.53 times more likely (95% CI: 1.14, 2.08; Table 3, Figure 3). Metrics for covariates are included in Table S1.

In nearly all cases, released sparrows that became breeders at Three Lakes did so during the first season post‐translocation. Only 4 Florida grasshopper sparrows were not confirmed as breeders until the second year after their release, yielding a total of 40 birds (15.1%) that recruited into the breeding population during our study (Table 1). An additional 14 grasshopper sparrows, nearly all males, were observed at least once in the study area over the 2‐year experiment but were never confirmed holding a breeding territory, and their ultimate fate was unknown.

## 4 Discussion

We found that a non‐migratory passerine raised in a conservation breeding facility demonstrated higher recruitment when translocated during its hatch‐year than when translocated during its second year after spending a winter in captivity. Despite being translocated only a few weeks before the onset of breeding, second‐year birds failed to settle and recruit into the breeding population at rates comparable to individuals translocated 6–8 months earlier as nutritionally independent fledglings, who subsequently had to survive the winter before reaching their first breeding season. A recent systematic literature review revealed that younger birds tend to survive better than older birds after they are translocated, but the impact of age of release on recruitment could not be assessed, primarily because most translocation experiments either did not measure or did not report those outcomes (Miller et al. 2024). Our experimental approach and intensive demographic monitoring of our color‐banded population at the recipient site enabled us to fill this information gap.

Holding juveniles in conservation breeding facilities through their first winter is a common practice because it is believed to increase survival by sheltering birds from periods of expected high mortality in the wild (Cristinacce et al. 2008; Cunninghame et al. 2015). However, extended care at a conservation breeding facility requires considerable time, space, and expense, and those costs should be weighed against the potential benefits. Potential detrimental consequences associated with the prolonged captivity of birds have been suggested, including potential dependence on captive food sources (Lagios et al. 2015), the development of tame behaviors (Buner et al. 2011), difficulty in adapting to harsh environmental conditions (Hardouin et al. 2014; Tripovich et al. 2021), and an inability to avoid wild predators (Buner and Schaub 2008; Rantanen et al. 2010). Birds released at younger ages have increased neural plasticity (Clark et al. 2023), and therefore a greater capacity and opportunity to learn behaviors necessary for survival in the wild (Crates et al. 2023).

Moreover, neural plasticity is a developmental phenomenon that extends to both wild‐caught and captive‐reared birds, as younger individuals are developmentally better suited to learn important skills (Vargas and Anderson 1999; Griffin et al. 2000) and adapt and settle into novel environments (Mathews et al. 2016; Wilcoxen et al. 2011). Experiments indicate that neophobia increases with age for many bird species (e.g., in corvids and parrots, O'Hara et al. 2017; and in a statistical learning model Sherratt and Morand‐Ferron 2018), which may intensify translocation‐related stress (Dickens et al. 2010) for older individuals. A negative effect of release age on post‐release survival has been found across a range of taxonomic groups, including waterfowl (Black et al. 1997; Green et al. 2005), gamebirds (Buner and Schaub 2008; Rantanen et al. 2010; Buner et al. 2011; Troy et al. 2013; Hardouin et al. 2014), and passerines (Bradley et al. 2012; Masuda and Jamieson 2012; VanderWerf et al. 2014; Lagios et al. 2015).

In our experiment, we were unable to determine the mechanisms that led to greater recruitment for Florida grasshopper sparrows released at younger ages, but a combination of factors could be involved, including accelerated social learning, exploration, and information gathering. Earlier releases allowed more time for sparrows to become familiar with the landscape, which could increase foraging efficiency and facilitate future territory establishment (Doligez et al. 2002; Betts et al. 2008). For Florida grasshopper sparrows, the post‐fledging period is a critical time for exploration outside the natal area (Small et al. 2015), and their forays may help them locate patches of recently burned prairie that are preferred for nesting (Shriver and Vickery 2001; Hewett Ragheb, Miller, and Kiltie 2019). Although our breeding season monitoring concluded before the end of the summer, we opportunistically observed released hatch‐year sparrows flocking with resident hatch‐year sparrows, which may have facilitated their settlement into the population and subsequent recruitment. Similarly, Swinnerton (2001) suggested that Pink Pigeons ( *Nesoenas mayeri* ) released at younger ages had greater survival because they were more likely to be accepted into flocks of wild birds and not chased off.

It remains unclear whether the low recruitment rate of captive‐bred sparrows released during their second year was the result of higher mortality or dispersal outside of our study site. Differences in the predator community composition at the site during the time of release may play a role. Birds released as hatch years had more time to acclimate to their new environment prior to the arrival of migratory raptors that winter at the site and are known to prey on Florida grasshopper sparrows (e.g., northern harriers [ *Circus cyaneus* ]; Dean 2001), in contrast to second‐year releases, which were released during the late winter when predators were already present in the area. Moreover, more time spent under managed care can be correlated with less responsiveness to wild predators (Carrete and Tella 2015). The relationship between the age of release and post‐translocation dispersal is not consistent across avian taxa (Miller et al. 2024), but for some species, translocation at older ages can result in greater dispersal, both for birds sourced from wild populations (Schadewinkel 2013; Sullivan 2006) and from conservation breeding facilities (Le Gouar et al. 2008). Grasshopper sparrows are known to disperse within and between breeding seasons (Williams and Boyle 2018, 2019), and Florida grasshopper sparrows have been detected dispersing between subpopulations (Tucker Jr et al. 2010; Hewett Ragheb et al. 2022). Dispersal off‐site may have occurred in our study, but members of the Florida Grasshopper Sparrow Working Group, who monitor Florida grasshopper sparrow populations on nearby properties, did not report any sightings of our released birds in the first year after their release.

Comparison with vital rates from wild populations can be useful when evaluating the success of captive‐breeding and release programs (Miller et al. 2024). Recruitment rates of captive‐reared Florida grasshopper sparrows released as nutritionally independent fledglings (21% and 16% during 2019 and 2020; Table 1) were comparable to recruitment rates of color‐banded wild fledglings in the ongoing sparrow demographic study (14% and 22% during the same years; Oteyza and Miller 2021), which provided further evidence that sparrows released as hatch‐years adapted and integrated into the local sparrow population. Moreover, our results compare favorably to recruitment rates in the literature for captive‐reared passerines, which tend to be < 10% after translocation (e.g., Nichols et al. 2010; Switzer et al. 2013; Charles Darwin Foundation 2018). Recruitment of sparrows released as hatch years in our study averaged 18% (Table 1), which is similar to the 23% recruitment rate reported for a reintroduced population of captive‐reared helmeted honeyeaters ( *Lichenostomus melanops cassidix* ; Menkhorst et al. 2010). We caution that recruitment rates may represent a conservative measure of a reintroduction program's overall success because populations of sparrows and other passerines are often male‐biased (Donald 2007), and therefore some individuals may survive but not breed.

We note that season and age are confounded in the design of most translocation studies (see figure 5 in Miller et al. 2024), and our study was no exception; i.e., we released juveniles during the summer and young adults during the late winter. We did not include a release date covariate in our analyses because releases were clustered by age into two distinct seasons in disjunct periods of the year. However, more data from ongoing releases of hatch‐years will help us better understand the relative importance of fledgling age and ordinal date *within* the summer season in relation to body condition and other attributes. Precise determination of temporal effects within a bird's first year of life may be difficult because relationships may not be linear (Bacon et al. 2019; Tripovich et al. 2021) and conservation breeding conditions may change over time (Fountain et al. 2017; Gosselin et al. 2025).

Implementation of effective translocation programs depends on several factors, including tradeoffs among budgetary, logistical, ecological, and political considerations (e.g., Converse et al. 2013; McCarthy et al. 2012), but releasing animals at their optimal age for survival and reproduction should increase the likelihood of any program's effectiveness (Letty et al. 2007; Miller et al. 2024). Based on results from this study, the Florida Grasshopper Sparrow Working Group has continued the conservation breeding and translocation program since 2022 using only hatch‐year releases. In the absence of site‐specific experiments like ours, conservation breeding programs may find that releasing younger ages may be a strategic choice (Miller et al. 2024). If younger age classes perform equally well, or better, than older age classes, then choosing younger birds for release also would enable the conservation breeding facility to keep producing more birds with less space and at less cost.

## Author Contributions

**Juan C. Oteyza:** conceptualization (supporting), data curation (lead), formal analysis (equal), investigation (equal), methodology (equal), project administration (equal), supervision (lead), writing – original draft (supporting), writing – review and editing (supporting). **Karl E. Miller:** conceptualization (equal), formal analysis (equal), funding acquisition (equal), investigation (supporting), methodology (supporting), project administration (supporting), writing – original draft (lead), writing – review and editing (lead). **Andrew Schumann:** conceptualization (equal), funding acquisition (supporting), methodology (equal), project administration (equal), writing – review and editing (supporting). **Sarah Biesemier:** data curation (supporting), investigation (supporting). **Andrea Sylvia:** formal analysis (lead), software (lead), writing – review and editing (supporting).

## Conflicts of Interest

The authors declare no conflicts of interest.

## Supporting information


**Table S1.** Summary table of captive‐reared Florida grasshopper sparrows ( *Ammodramus savannarum floridanus* ) translocated to Three Lakes Wildlife Management Area, FL, USA in 2019 and 2020. Variables comprise those used in the logistic regression models (HY = hatch‐year juveniles, SY = second‐year adults).

Data S1.

## Data Availability

All the required data are uploaded as [Link], [Link].
