# Supplementary material for: Recruitment of Captive‐Reared Florida Grasshopper Sparrows After Translocation: Age of Release Matters
Source: Ecol Evol. 2025 Jun 26;15(7):e71662. doi: 10.1002/ece3.71662 (PMC12202780; doi:10.1002/ece3.71662)
Supplement: Supplementary file 1 — Table S1. Summary table of captive‐reared Florida grasshopper sparrows ( Ammodramus savannarum floridanus ) translocated to Three Lakes Wildlife Management Area, FL, USA in 2019 and 2020. Variables comprise those used in the logistic regression models (HY = hatch‐year juveniles, SY = second‐year adults). [file ECE3-15-e71662-s001.docx]

Appendix Table S1. Summary table of captive-reared Florida grasshopper sparrows (*Ammodramus savannarum floridanus*) translocated to Three Lakes Wildlife Management Area, FL, USA in 2019 and 2020. Variables comprise those used in the logistic regression models (HY = hatch year juveniles, SY = second-year adults).

| Release age class | Recruit | Cohort 2019 | Cohort 2020 | Male | Female | Mass mean (SE) | Mass min-max | Fat score mean (SE) | Fat score min-max |
| --- | --- | --- | --- | --- | --- | --- | --- | --- | --- |
| HY | Yes | 16 | 16 | 17 | 15 | 16.4 (0.2) | 14.0 – 18.6 | 2.1 (0.2) | 0 – 4 |
|  | No | 62 | 87 | 77 | 72 | 16.1 (0.1) | 13.3 – 18.5 | 2.0 (0.1) | 0 – 4 |
| SY | Yes | 0 | 4 | 3 | 1 | 18.3 (1.1) | 15.6 – 20.3 | 2.3 (0.3) | 2 – 3 |
|  | No | 38 | 42 | 48 | 32 | 17.1 (0.1) | 15.2 – 21.0 | 2.1 (0.1) | 1 – 4 |
